# Combining multiple ChIP-seq peak detection systems using combinatorial fusion

**DOI:** 10.1186/1471-2164-13-S8-S12

**Published:** 2012-12-17

**Authors:** Christina Schweikert, Stuart Brown, Zuojian Tang, Phillip R Smith, D Frank Hsu

**Affiliations:** 1 Laboratory for Informatics and Data Mining, Department of Computer and Information Science, Fordham University, New York, NY 10023, USA; 2 Center for Health Informatics and Bioinformatics, New York University Langone Medical Center, New York, NY 10016, USA

## Abstract

**Background:**

Due to the recent rapid development in ChIP-seq technologies, which uses high-throughput next-generation DNA sequencing to identify the targets of Chromatin Immunoprecipitation, there is an increasing amount of sequencing data being generated that provides us with greater opportunity to analyze genome-wide protein-DNA interactions. In particular, we are interested in evaluating and enhancing computational and statistical techniques for locating protein binding sites. Many peak detection systems have been developed; in this study, we utilize the following six: CisGenome, MACS, PeakSeq, QuEST, SISSRs, and TRLocator.

**Results:**

We define two methods to merge and rescore the regions of two peak detection systems and analyze the performance based on average precision and coverage of transcription start sites. The results indicate that ChIP-seq peak detection can be improved by fusion using score or rank combination.

**Conclusion:**

Our method of combination and fusion analysis would provide a means for generic assessment of available technologies and systems and assist researchers in choosing an appropriate system (or fusion method) for analyzing ChIP-seq data. This analysis offers an alternate approach for increasing true positive rates, while decreasing false positive rates and hence improving the ChIP-seq peak identification process.

## Background

### Introduction

One of the most important biotechnologies developed in the 20^th ^century is the Sanger method for the sequencing of DNA [1]. Recently developed next-generation DNA sequencing (NGS) technologies have increased DNA sequencing capacity by many orders of magnitude, making entirely new applications possible [2,3]. **Ch**romatin **I**mmuno**P**recipitation (**ChIP**) is a biochemical method to identify binding sites on DNA that interact with proteins. It involves cross-linking proteins to DNA with a reagent such as formaldehyde, randomly shearing the DNA into small fragments (200-500 base pairs) (**fragmentation**), then using an antibody specific for a known DNA-interacting protein to isolate DNA fragments bound to the target protein [4] (**immunoprecipitation**).

**Figure 1:**
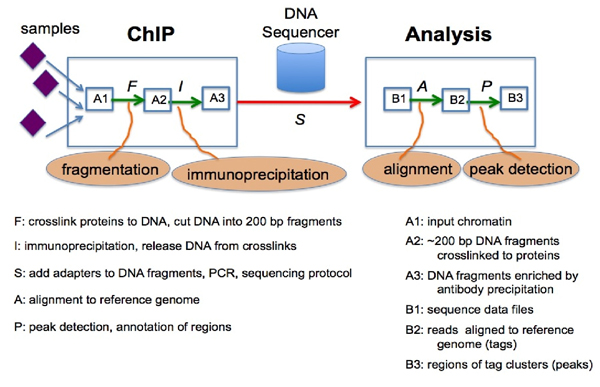
**ChIP-seq experiment and analytic workflow**.

The combination of the ChIP process and microarray DNA chip technologies lead to the method of Chip-on-chip [5] or **ChIP-chip **[6] that can identify DNA fragments isolated by ChIP using a DNA microarray containing large numbers of probes of known genomic sequences. **ChIP-seq **[7] uses next-generation sequencing (NGS) to identify the DNA fragments isolated by ChIP. Next-generation DNA sequencing machines are capable of simultaneously determining the sequences of millions of DNA fragments in a single sample with a high degree of accuracy (**high-throughput sequencing**). The sequence reads (known as tags) obtained from ends of ChIP-selected DNA fragments are typically 25-50 base pairs long. These short reads can then be mapped to a reference genome by a stringent DNA sequence alignment algorithm such as ELAND (Illumina Inc.), MAQ [8], or Bowtie [9] (**mapping**). Sequence reads that do not map to a unique position on the genome (with 2 or fewer mismatches) are generally discarded. The final product of such a mapping procedure is a set of positions on the reference genome indicating the start and end of each short sequence read. Once the reads are mapped to the genome, the tag positions can then be analyzed for clusters of tags or "peaks", which indicate (predict) protein binding (or histone modification) positions enriched by the ChIP (**peak detection**). The results of ChIP-seq studies can provide an unbiased genome-wide profile of DNA regulatory regions targeted by transcription factors as well as the signatures of modified histone proteins associated with epigenetic changes in chromatin. Figure 1 shows a framework for a ChIP-seq experiment and analytic workflow [10].

Peak detection is the last and probably most crucial and dynamic step in the process of the ChIP-seq method and system after fragmentation, immunoprecipitation, sequencing, and mapping. Along the pipeline, the set of mapped sequence tags can easily acquire noise from background contamination, co-precipitation of unbound DNA fragments, non-specific interactions of the ChIP target protein with DNA, and a variety of sources such as replication and amplification artifacts (e.g. PCR artifacts). A useful ChIP-seq peak detection technique or tool has to be robust and reliable. With the rising popularity and increasing importance of ChIP-seq, there has been a proliferation of new analytical and computational methods to find peaks in ChIP-seq data. At the last count, there are over 30 open source programs, in addition to many commercial software applications, available to the research community [11].

The first step in the peak detection process is to identify those genomic regions with a large number of mapped sequence tags (enriched regions) [12-24]. Then the peak detection and identification system must determine the number of tags (peak heights) or directionality score (**tag count) **that constitutes enrichment "significant" enough to represent a protein-DNA binding site. In this way, tag count (**T**) is a scoring function in which the system assigns a number to each possible region. Often, a tag count threshold is chosen to define a peak [24]. One way to set this threshold is to compare the distribution of tags in enriched regions to tags that are placed randomly on the genome. The outcome is a significance value (**p-value**) of the sequence tag enrichment. This value (**P**) is also a scoring function used to select peaks [16,17,20,22]. Some methods use sequence data from a control dataset and then use the control tag densities to assess the significance of peaks in the ChIP sample set. In this case, a **fold enrichment **(**F**) ratio of ChIP tag count over the normalized control tags in the candidate regions is calculated to give another scoring function [7,14,17,25]. Different methods use various statistical models to assess the significance of ChIP peaks or assign a false discovery rate (FDR) to each peak with respect to control data [12,16,18-26].

Recently, Pepke et al [27] gave a review of the major steps in ChIP-seq analysis and described the algorithmic approaches of twelve existing programs for detecting peaks. Laajala et al [28] gave some metrics for evaluating various methods of peak detection. Wilbanks and Facciotti [11] compared the performance of eleven different peak calling programs on common empirical, transcription factor data sets. Their work offers a variety of ways to assess the performance of each algorithm and address the questions as to how to select the most suitable among several available methods of ChIP-seq analysis for peak detection. In our study, we evaluate six methods: CisGenome, MACS, PeakSeq, QuEST, SISSRs, and TRLocator [16,17,23,24,26,29] based on the three attributes: tag count, p-value, and fold change, and their combinations. We then analyze the merged results of all two-method combinations. In particular, a recently developed information fusion method, Combinatorial Fusion Analysis [30], is utilized to demonstrate that ChIP-seq peak detection can be improved by fusion using score or rank combination. Our study offers an alternate approach to select a suitable method for ChIP-seq analysis. This study also offers ways to improve existing methods by combining them in an appropriate way using Combinatorial Fusion Analysis.

Based on preliminary experiments, we have observed that the peak-detection abilities of available ChIP-seq methods and systems vary greatly depending on the type of protein that is targeted by the antibody used in the ChIP. We have identified three types of protein-DNA interactions that generate very different results when the same peak detection system is used to analyze the ChIP-seq data [10]. The first observation is that transcription factors, such as E2F4, bind strongly to a single highly specific DNA sequence (a motif) near the transcription start site (TSS) of a gene, and are characterized by distinct ChIP-seq peaks ~500 bases wide, with oriented tags that approximately follow a normal distribution. A second observed pattern is with transcription factors, such as Sin3a, that bind weakly to DNA together with co-factors, yielding wider ChIP-seq peaks (800-1600 bases) with a flat distribution of lower tag density and un-oriented tags. A third kind of ChIP-seq target, modified histone proteins, such as tri-methylated H3K4, produce much wider peaks (~4000 bases) and un-oriented tags [10]. In this study, we use a trimethylated H3K4 (H3K4me3) data set [31].

### Previous work

Similar to the analysis of microarray gene expression data, many computational methods have recently been developed for the analysis of ChIP-seq data. In both cases, the proliferation of software and systems was an indication that it is difficult to find a single well-validated method that performs well in a variety of domain applications. It also depends on what criteria one uses to evaluate the systems. In this study, we use the following six methods and systems to analyze their intra- and inter-system properties and improvement by combination. They are (A) CisGenome [16], (B) MACS [24], (C) PeakSeq [26], (D) QuEST [23], (E) SISSRs [17], and (F) TRLocator [29].

CisGenome [16] uses a two-pass algorithm for peak detection to ensure adjustment for DNA fragmentation length. It can analyze both ChIP-seq and ChIP-chip data, or combine the two. In order to correct many types of systemic bias created by sample preparation, amplification, sequencing (or hybridization), and alignment, it uses both a ChIP sample and a negative control sample (input DNA or mock-ChIP with IGG) to compute FDR at each specific location. It also provides methods to detect binding regions, peak localization, and filtering.

QuEST [23] provides a data-driven statistical analysis model to generate peak calls by leveraging the key attributes of the sequenced and aligned DNA reads, such as directionality (strand orientation) and the original size of ChIP-isolated DNA fragments. The statistical framework used is the kernel density probability estimation approach, which facilitates the aggregation of signals originated from densely packed sequence reads at protein interaction sites.

MACS (Model-based Analysis of ChIP-Seq) [24] empirically models the shift size of ChIP-seq tags to enhance peak identification by taking advantage of the bimodal pattern of forward and reverse tags. MACS also utilizes a dynamic Poisson distribution to identify local biases in the genome.

Site Identification from Short Sequence Reads (SISSRs) [17] estimates high read counts using Poisson probabilities and calls regions where the peaks shift from the forward to the reverse strand. The SISSRS method is attractive because it explicitly makes use of information from the orientation of tags around a protein binding site - where it is expected that forward strand tags will be found upstream of the true binding site and reverse strand tags downstream. This allows for very precise prediction of the actual binding site. However, for regions of low tag density or for histone methylation ChIP, where tags are not neatly oriented, it tends to create many different peaks across enriched regions, which may not be reproducible across replicates.

PeakSeq [26] utilizes input-DNA control data to refine the selection and scoring of peak regions in Chip-seq experiments to improve the identification of transcription factor binding sites. Since it has been observed that signal peaks in the control data are highly correlated with potential binding sites, PeakSeq compensates for this signal, caused by open chromatin structure, with a two-pass strategy. PeakSeq first identifies enriched peaks in the Chip-seq data as candidate regions. These putative regions are then compared to the normalized control and the regions that are significantly enriched with mapped sequence tags relative to the control are identified as binding sites.

TRLocator [29] is a peak detection method that has been developed at NYU-CHIBI. The algorithm utilizes the distribution of the background data to compute p-values for putative peaks in the ChIP-seq data. Putative peak regions are generated based on a variable merging window size that can be adjusted according to the kind of data set being analyzed. Custom filters for finding qualified peak regions include: p-value, minimum number of tags within each putative peak, balance between the number of tags aligned to the positive strand and the number of tags aligned to the negative strand, and the log2 ratio between ChIP tags and background tags.

## Methods

### Combining peak detection systems

#### Multiple scoring systems

We propose that the peak detection for each of the binding sites be viewed as a scoring system on the set of all possible binding site regions. Different scoring systems for peak detection can represent different features/cues/attributes or different algorithms/methods/systems. They can also represent different technical replicates or different biological replicates using each of the same set of features or cues/attributes or the same algorithm or method/system. By using multiple scoring systems defined on the set of possible binding site regions to detect peaks for each of the binding sites, we can study the reproducibility of peak calls among different replicates. We also use multiple scoring systems to develop and design new algorithms with greater accuracy, efficiency, and scalability for detecting protein binding sites in ChIP-seq data alignment. We draw from recent research in combinatorial fusion [32,33]. Using a rank-score characteristic graph to measure the scoring diversity [34], combinatorial fusion has been an active research area in the past ten years in a variety of application domains such as microarray gene expression analysis [35], motif finding [36], protein structure prediction [37], virtual screening [38], information retrieval [39,33], and target tracking [40].

In our preliminary work, we analyzed the six individual systems according to three features, which include: tag count, p-value, and fold change (enrichment of ChIP tags compared to background control tags at the same genomic locus) [41]. We analyzed these features and their combinations according to average precision and observed that, in most cases, the tag count feature outperformed other features and combinations of features. Since tag count was the most consistent and best performing feature between the methods, we choose to use the ChIP tag count as the score function to represent each method's scoring of the regions identified. Let *D *= *{d_1_, d_2_,..., d_n_} *be the set of regions identified by system x and the score function *s*_x_*(d) *be the tag count of that region (number of ChIP tags in the data set that are located within that chromosomal region). Let the rank function *r*_x_*(d) *be the function from *D *to *N = *[*1, n*] *= {1,2,..., n} *which is obtained by sorting the values in *s*_x_*(d) *into descending order and converting the function *s*_x_(*d*) into the function *r*_x_(*d*) using the rank as its function value.

### Combining two peak detection systems

#### Union

The union of two systems, x and y, U(x, y) is the set of regions that contains all regions identified by x and all regions identified by y, where overlapping regions between the two methods are merged together to form new merged regions. All non-overlapping regions that belong to either x or y will maintain their genomic positions (chromosome, start and end bp coordinates). Each merged region will have a start position that is the minimum of all start positions of its overlapping regions from x and y, and an end position that is the maximum of all end positions of those overlapping regions. This new set of regions, U(x, y) = *{d_1_, d_2_, ..., d_p_}*, is scored based on the tag counts of systems x and y, as follows. Systems x and y have new score functions based on the regions in this union: *s*_x'_(*d*) and s_y'_(*d*).

*s*_x'_(*d*) is obtained according to the following:

Single regions - if the region was identified by system x, the score is the tag count given by x; otherwise the score is 0.

Merged regions - the score is the sum of the tag counts for the regions (that are part of this merged region) that were identified by x.

*s*_y'_(*d*) is obtained in the same manner. The score functions are then scaled from 0 to 1 by the following normalization: score function *s*_x'_(*d*)*: U(x, y) → R *is transformed to

$$s_{{\text{x}}^{\prime }}*\left(d\right):U\left(x,y\right)\to \;\left[0,1\right]\;\text{where,}\;s_{{\text{x}}^{\prime }}*\left(d\right)=\frac{s_{{\text{x}}^{\prime }}\left(d\right)-s_{\text{min}}}{s_{\text{max}}-s_{\text{min}}},$$

*s_max _= max{ s*_x'_(*d*): *d*∈*U(x, y)}*, and *s_min _= min{ s*_x'_(*d*)*: d*∈*U(x, y)}. s*_y'_(*d*) is also normalized accordingly. The rank functions *r*_x'_(*d*) and *r*_y'_(*d*) from *U(x, y) *to *N = {1, 2, ..., p} *assign a rank to each region after sorting the scores given by *s*_x'_(*d*) and *s*_y'_(*d*) in descending order, respectively. In order to provide a single score and rank for each region in U(x, y) that is based on combined information from systems x and y, we perform score and rank combinations. The score combination for the union of systems x and y is defined as:

$s_{U\left(x,y\right)}\left(d\right)=\frac{1}{2}\left(s_{{\text{x}}^{\prime }}*\left(d\right)+s_{{\text{y}}^{\prime }}*\left(d\right)\right)$and the rank combination is computed according to:

$$r_{\text{U}\left(\text{x,y}\right)}\left(d\right)=\frac{1}{2}\;\left(r_{{\text{x}}^{\prime }}\left(d\right)+r_{{\text{y}}^{\prime }}\left(d\right)\right).$$

#### Intersection

The intersection of two systems, x and y, I(x, y) is the set of the merged regions formed by overlapping regions of system x and y. I(x, y) ⊆ U(x, y) where I(x, y) = *{i ∈ U(x, y): i is a merged region that contains overlapping regions from both systems x and y}*, giving the set I(x, y) = *{d_1_, d_2_, ..., d_q_}*. The regions belonging to the intersection are scored in the same way merged regions are scored in the union. The score functions for systems x and y, based on their intersection, *s*_x'_(*d*) and s_y'_(*d*), assign a score to each of the merged regions that is the sum of the tag counts for the regions identified by x or y that are part of this merged region.

*s*_x'_(*d*) and s_y'_(*d*) are then normalized to the scale [0,1] (as described above) to give *s*_x'_*(*d*) and s_y'_*(*d*). The regions of the intersection are ranked according to their score (descending order) to give rank functions *r*_x'_(*d*) and *r*_y'_(*d*). Similar to the case of union, score and rank combinations for the intersection of systems x and y are computed. The score and rank combinations are defined as: $s_{\text{I}\left(\text{x,y}\right)}\left(d\right)=\frac{1}{2}\;\left(s_{{\text{x}}^{\prime }}*\left(d\right)+s_{{\text{y}}^{\prime }}*\left(d\right)\right)$ and $r_{\text{I}\left(\text{x,y}\right)}\left(d\right)=\frac{1}{2}\;\left(r_{{\text{x}}^{\prime }}\left(d\right)+r_{{\text{y}}^{\prime }}\left(d\right)\right)$, respectfully.

**Figure 2:**
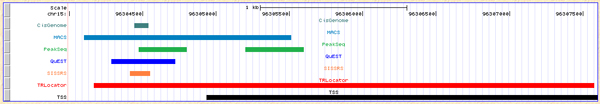
**Regions of individual methods upstream of the ARRDC4 gene**.

**Figure 3:**
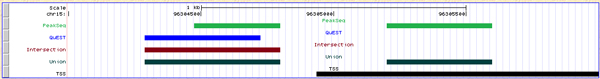
**Example of the intersection and union between PeakSeq and QuEST regions**.

Example from H3K4 data set: The visualization in Figure 2 shows peaks identified by all individual methods, along with the TSS regi2on, near the ARRDC4 gene **(ARRDC4; Chromosome: 15; 96,304,937-96,318,072, UCSC Genome Browser Mar. 2006 assembly)**. Figure 3 demonstrates the intersection and union of the PeakSeq and QuEST methods in the area depicted above. The intersection contains the merged regions that are formed by overlapping regions between the two methods. The union contains these merged regions and all non-overlapping regions of the individual methods.

### Performance evaluation methods

#### Average precision

**Figure 4:**
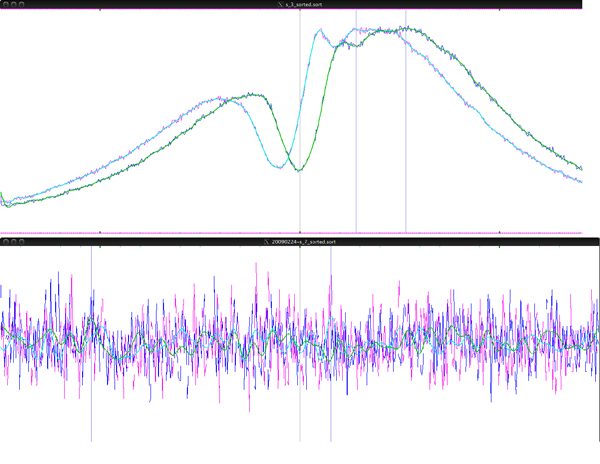
**ChIP-seq tags from an immunoprecipitation with antibody for H3K4 and an IGG control**. The graph shows the total number of tag start positions mapped to each basepair within 1000 bp flanking all annotated RefSeq TSS. Tags mapped to the forward strand are shown in blue and the reverse strand in green. The graph also shows a very clear nucleosome depleted region located exactly at the TSS.

For many transcription factors, DNA polymerase II, and some modified histones such as tri-methylated H3K4, the majority of binding sites are located near the transcription start sites (TSS) of expressed genes. Therefore, it is possible to evaluate ChIP-seq software systems, and different combination methods, by their ability to locate peaks at a TSS. While not all true peaks are located at a TSS, not all TSS are correctly annotated in the reference genome, and not all true TSS have such a peak, the ratio of peaks located at an annotated TSS vs. those located elsewhere on the genome is a measure of precision of the peak finding method. We have validated this concept by visualizing all aligned tags on the genome without first identifying peaks. Peaks can be observed in the vicinity of most TSS annotated in the RefSeq database. An average peak can be visualized by superimposing the coverage depth of sequence reads for DNA regions within 1000 bases flanking all annotated RefSeq TSS (Figure 4). No TSS peak is found in control DNA.

In this evaluation, we compare the peaks identified by a particular system (or combination of two systems) against the set of RefSeq TSS in the human genome. Average precision is used to evaluate the performance of systems and the result of fusion. A region is considered relevant if it overlaps with a TSS in the annotated set. We define the following overlap function for a region at rank *i*:

$$o\left(i\right)=\left\{\begin{array}{c} 1,\;region\;overlaps\;with\;a\;TSS\\ 0,\;otherwise \end{array}\right)$$

Precision at rank *r *is computed as:

$$p\left(r\right)=\frac{\overset{r}{\underset{i=1}{\sum }}o\left(i\right)}{r}$$

Average precision for a system that identifies *n *regions is defined as:

$$AP\left(n\right)=\frac{\overset{n}{\underset{r=1}{\sum }}\left(p\left(r\right)\times o\left(r\right)\right)}{n}.$$

Systems that have more regions in lower ranks that overlap with a TSS will have higher average precision.

#### Coverage

When evaluating the performance of a peak finding system, it is also important to consider its coverage, in terms of the breadth of TSS covered by its peaks (regions). Given a set of regions identified by a system or combination method, we generate the set of TSS that overlap with these regions; the coverage (C) is the number of *unique *TSS reached by the system:

$$C\;=\;\left|\left\{TSS\;that\;overlap\;with\;region\left(s\right)\;of\;system\right\}\right|.$$

## Results

### System fusion and evaluation

**Figure 5:**
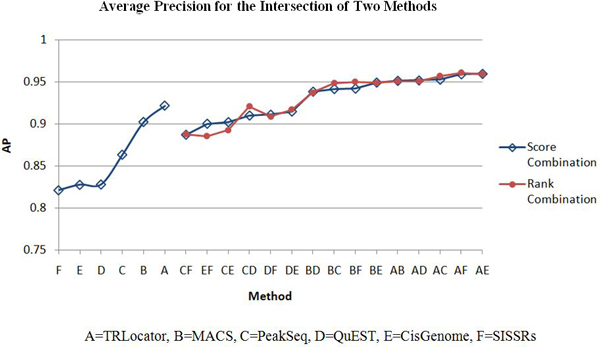
**Average precision for intersection of two methods**.

**Figure 6:**
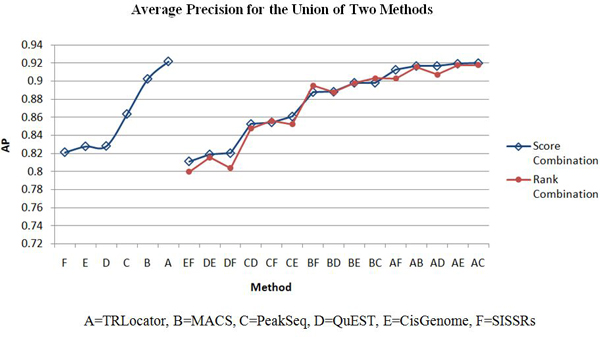
**Average precision for union of two methods**.

**Figure 7:**
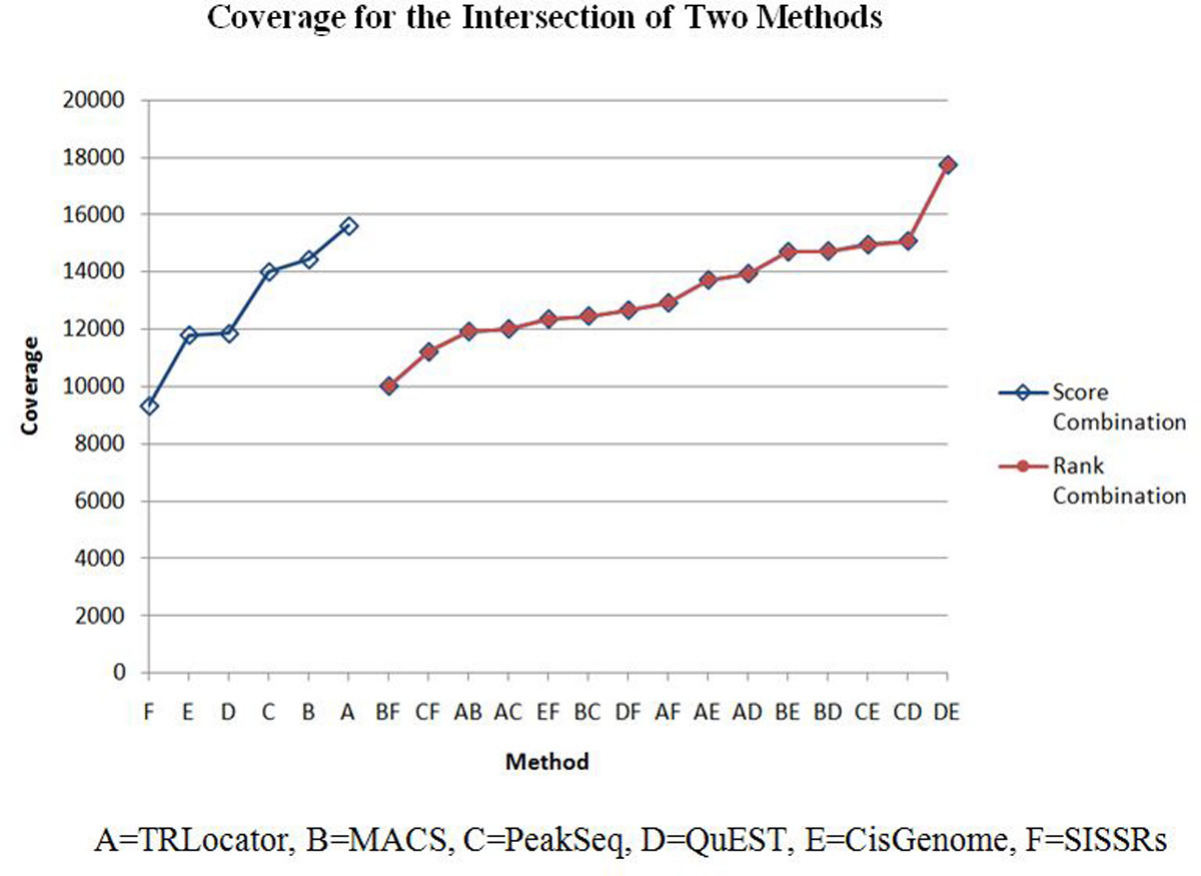
**Coverage for intersection of two methods**.

**Figure 8:**
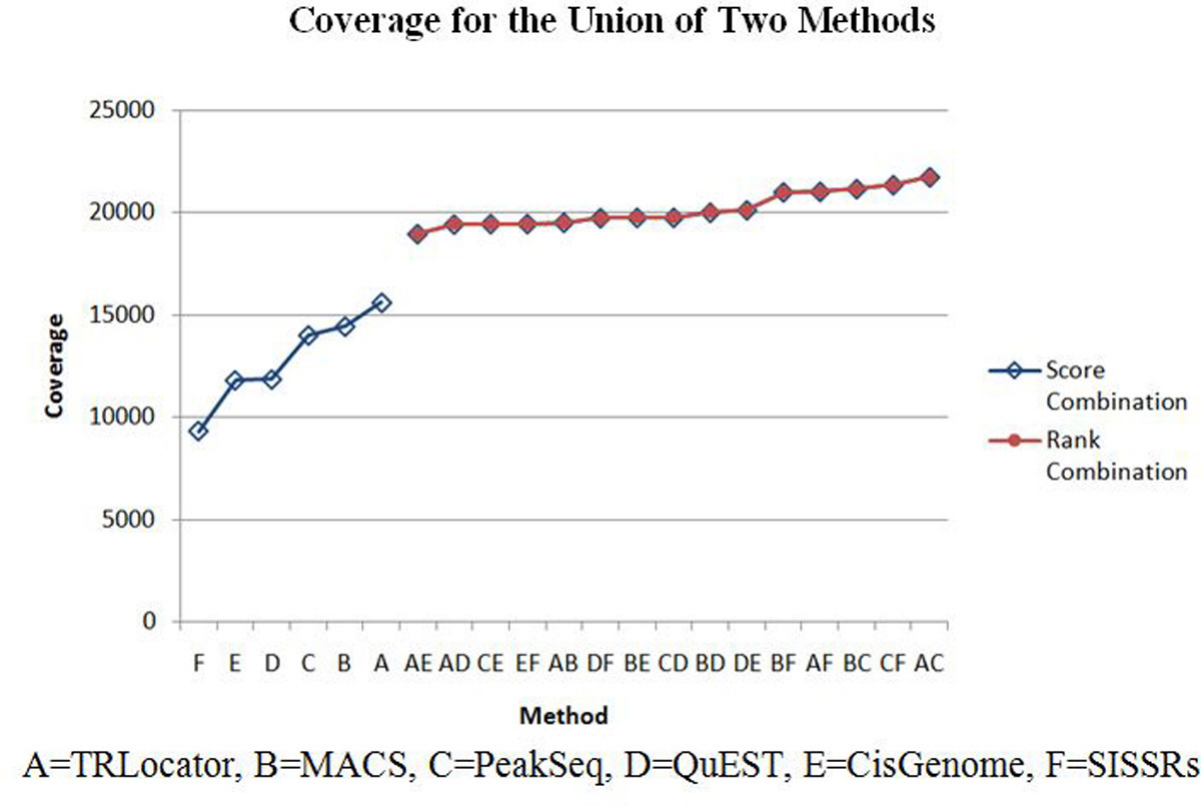
**Coverage for union of two methods**.

The ChIP tag count for a region is used as a score function to create all 2-combinations of the six systems: CisGenome, MACS, PeakSeq, QuEST, SISSRs, and TRLocator. We perform two kinds of combination: intersection and union (see Methods section). The intersection of two systems is expected to improve specificity (detected by both systems) while the union is expected to improve sensitivity (detected by either system). When evaluating each system or combination of two systems, we use average precision and coverage (see Methods section). These results are listed in Tables 1, 2, 3, 4, 5, 6 with corresponding diagrams in Figures 5, 6, 7, 8.

**Table 1:** Average precision for single methods.

Method	Average precision; rank	Number of regions
F = SISSRs	0.8212; 6	20715
E = CisGenome	0.8277; 5	21190
D = QuEST	0.8281; 4	21514
C = PeakSeq	0.8634; 3	20000
B = MACS	0.9023; 2	19918
A = TRLocator	0.9217; 1	19673

**Table 2:** Average precision for the intersection (*) of two methods.

x * y	Average precision		Number of regions
		
	Score combination	Rank combination	
C * F = PeakSeq * SISSRs	0.887166	0.887675	13293
E * F = CisGenome * SISSRs	0.900260	0.885596	12841
C * E = PeakSeq * CisGenome	0.902211	0.892652	12662
C * D = PeakSeq * QuEST	0.910056	0.920872	11865
D * F = QuEST * SISSRs	0.911046	0.908774	10789
D * E = QuEST * CisGenome	0.914799	0.917028	14452
B * D = MACS * QuEST	0.938479	0.937476	14528
B * C = MACS * PeakSeq	0.941655	0.948495	12095
B * F = MACS * SISSRs	0.942113	0.950036	11003
B * E = MACS * CisGenome	0.949365	0.948955	14244
A * B = TRLocator * MACS	0.951392	0.950802	16921
A * D = TRLocator * QuEST	0.951877	0.950939	13270
A * C = TRLocator * PeakSeq	0.952759	0.956961	11573
A * F = TRLocator * SISSRs	0.959214	0.960584	10463
A * E = TRLocator * CisGenome	0.959687	0.959111	13155

**Table 3:** Average precision for the union (+) of two methods.

x + y	Average precision		Number of regions
		
	Score combination	Rank combination	
E + F = CisGenome + SISSRs	0.8114	0.7997	26371
D + E = QuEST + CisGenome	0.8190	0.8158	26457
D + F = QuEST + SISSRs	0.8204	0.8038	25574
C + D = PeakSeq + QuEST	0.8526	0.8475	22191
C + F = PeakSeq + SISSRs	0.8545	0.8559	22876
C + E = PeakSeq + CisGenome	0.8610	0.8522	24415
B + F = MACS + SISSRs	0.8880	0.8950	20767
B + D = MACS + QuEST	0.8883	0.8876	21242
B + E = MACS + CisGenome	0.8983	0.8977	20768
B + C = MACS + PeakSeq	0.8983	0.9033	19895
A + F = TRLocator + SISSRs	0.9126	0.9030	19673
A + B = TRLocator + MACS	0.9168	0.9158	20279
A + D = TRLocator + QuEST	0.9168	0.9071	20117
A + E = TRLocator + CisGenome	0.9193	0.9178	19720
A + C = TRLocator + PeakSeq	0.9199	0.9177	19281

**Table 4:** Coverage for single methods.

Method	Coverage; rank	Number of regions
F = SISSRs	9322; 6	20715
E = MACS	11804; 5	19918
D = TRLocator	11850; 4	19673
C = CisGenome	14010; 3	21190
B = QuEST	14440; 2	21514
A = PeakSeq	15611; 1	20000

**Table 5:** Coverage for the intersection (*) of two methods.

x * y	Coverage		Number of regions
		
	Score combination	Rank combination	
B * F = QuEST * SISSRs	10016	10016	10789
C * F = CisGenome * SISSRs	11211	11211	12841
A * B = PeakSeq * QuEST	11920	11920	11865
A * C = PeakSeq * CisGenome	12010	12010	12662
E * F = MACS * SISSRs	12351	12351	11003
B * C = QuEST * CisGenome	12459	12459	14452
D * F = TRLocator * SISSRs	12662	12662	10463
A * F = PeakSeq * SISSRs	12921	12921	13293
A * E = PeakSeq * MACS	13717	13717	12095
A * D = PeakSeq * TRLocator	13939	13939	11573
B * E = QuEST * MACS	14700	14700	14528
B * D = QuEST * TRLocator	14725	14725	13270
C * E = CisGenome * MACS	14947	14947	14244
C * D = CisGenome * TRLocator	15075	15075	13155
D * E = TRLocator * MACS	17725	17725	16921

**Table 6:** Coverage for the union (+) of two methods.

x + y	Coverage		Number of regions
		
	Score combination	Rank combination	
A + E = PeakSeq + MACS	18964	18964	19895
A + D = PeakSeq + TRLocator	19433	19433	19281
C + E = CisGenome + MACS	19458	19458	20768
E + F = MACS + SISSRs	19459	19459	20767
A + B = PeakSeq + QuEST	19520	19520	22191
D + F = TRLocator + SISSRs	19742	19742	19673
B + E = QuEST + MACS	19760	19760	21242
C + D = CisGenome + TRLocator	19767	19767	19720
B + D = QuEST + TRLocator	20014	20014	20117
D + E = TRLocator + MACS	20127	20127	20279
B + F = QuEST + SISSRs	21003	21003	25574
A + F = PeakSeq + SISSRs	21032	21032	22876
B + C = QuEST + CisGenome	21165	21165	26457
C + F = CisGenome + SISSRs	21360	21360	26371
A + C = PeakSeq + CisGenome	21738	21738	24415

Tables 1, 2, and 3 list the average precision for the six individual systems and all fifteen 2-combinations by intersection (*) and union (+). The six individual systems in order of performance according to average precision are: A = TRLocator, B = MACS, C = PeakSeq, D = QuEST, E = CisGenome, and F = SISSRs. In Table 2, it can be observed that all 2-combinations by intersection are positive cases, which means its performance is better than or equal to the best of the two individual systems. Each of the two combinations: A*E between TRLocator (A) (ranked #1) and CisGenome (E) (ranked #5) and A* F between TRLocator (A) (ranked #1) and SISSRs (F) (ranked #6) is better than the three 2-combinations A*B, A*C, and A*D that involve TRLocator (A) (ranked #1), MACS (B) (ranked #2), PeakSeq (C) (ranked #3), and QuEST (D) (ranked #4). Moreover, each of the 2-combinations B*E and B*F is better than the other 2-combinations B*C and B*D. This phenomenon is quite interesting - individual systems such as CisGenome (E) (ranked #5) and SISSRs (F) (ranked #6), which are lesser preferred, can be combined with other systems (in this case, with TRLocator (A) (ranked #1)) to outperform other system combinations. Almost all of the 2-combinations by union (+) in Table 3 are negative cases - the performance of the 2-combination is less than the best performance of the single cases - except for the 2-combination of B+C (MACS and PeakSeq). It is also interesting to note that the three 2-combinations A+C, A+D, and A+E are better than the 2-combination A+B, reflecting the same phenomenon observed in Table 2.

Tables 1, 2, 3, 4, 5, 6 list the four cases of inter-system fusion: average precision for 2-combinations by intersection and by union; and coverage for 2-combinations by intersection and by union. The huge difference between average precision of the intersection and union is that the former has all the positive 2-combination cases, while the latter has all (but one) negative cases. Comparing Table 5 and Table 6 we find that each of the unions of two systems in Table 6 has higher coverage than those of the intersections of two systems in Table 5. Another difference is that in Table 5, 2-combinations C*D, C*E, and D*E move up to the second, third, and first ranks, while in Table 6, the 2-combinations involving CisGenome (C) (ranked #3) and SISSRs (F) (ranked #6), such as A+C, C+F, B+C, A+F, and B+F move up to the top five rankings.

## Discussion

Evaluation of peak detection systems involves analyzing the regions identified as peaks according to criteria such as the average precision and TSS coverage.

Average precision measures the performance of a system according to higher scoring regions overlapping with a TSS. The intersection of two methods refers to the set of regions formed by extracting overlapping regions between two methods and merging them to form new regions. This set of regions represents the common peaks detected by both systems. The average precision of all 15 2-combinations improved when the intersection was evaluated. Combination by union only produced one result that improved average precision, MACS and PeakSeq.

When evaluating system combination according to TSS coverage, we refer to the number of unique TSS regions reached. When using the method of union to combine, all 15 2-combinations show improvement from both original systems. The result of combining two methods by union includes all overlapping regions that are then merged (intersection), in addition to all other regions belonging to each individual method. Some combinations show more improvement than others, which indicates that regions generated by those 2 systems are more diverse in terms of region location. For example, the regions identified by CisGenome overlap with 14010 unique TSS, and the coverage of PeakSeq is 15611. The combination of CisGenome and PeakSeq by union yields results that have a coverage of 21738, which means the combined result reaches many more TSS. Another example is for MACS and TRLocator, which individually have similar performance for coverage, 11804 and 11850, respectively. However, the combination of MACS and TRLocator by union greatly improves the performance and now reaches 20127 unique TSS; this demonstrates the diversity of the two systems. When using the method of intersection for system combination, 4 out of 15 combinations outperformed their component individual systems. Since the intersection consists of the merged, overlapping regions of two methods, improvement would take place if the merged region reaches a TSS missed by the regions before being merged.

## Conclusions

This study entails the evaluation of and selection among multiple detection systems for ChIP-seq peak identification. In order to do so, we use six well-known methods A = CisGenome, B = MACS, C = PeakSeq, D = QuEST, E = SISSRs, and F = TRLocator and obtain the regions identified by each on a common ChIP-seq data set and utilize the tag count as a score function representing each method. We define two methods to combine and rescore the regions of two systems, namely, union and intersection. Average precision and TSS coverage are used to evaluate the performance of all 2-combinations of these six systems. We summarize our results as follows:

(1) Average precision of intersection: All 2-combinations are positive cases

(2) Average precision of union: All 2-combinations (except one) are negative cases.

(3) Coverage of intersection: Some 2-combinations are positive, while some are negative.

(4) Coverage of union: All 2-combinations are positive cases.

(5) In the case of coverage of intersection, 2-combinations D*E, C*D, and C*E are ranked #1, #2, and #3 among all 15 2-combinations, respectively. For the coverage of union, 2-combinations A+C, C+F, B+C, A+F, and B+F are ranked #1, #2, #3, #4, and #5 among the 15 2-combinations, respectively.

In summary, we have the following observations resulting from the above experiments:

• There is no single answer as to the selection of available methods (and systems) for ChIP-seq peak detection. It depends on the criteria (e.g. features) and performance evaluation (e.g. average precision or TSS coverage).

• Combinations of different methods (systems) do improve results in many cases (average precision of intersection, coverage of union, some for coverage of intersection). Some combinations of lesser preferred systems may outperform all other system combinations.

• Average precision improved more when combining two systems by intersection and coverage improved more when two methods are combined by union.

• The use of the rank function in our evaluation of multiple detection systems provides a generic framework to study the preference and relative preference for the method (or system) selection process.

In our future work, we will explore conditions such as diversity between or performance ratio of two methods (systems) of which two or more systems should be combined to obtain a better system (positive cases). Future work also involves application of method combination to other proteins and transcription factors. As not all TSS may be annotated in the reference genome, identifying high-scoring regions among multiple methods can also be used to suggest potential TSS.

## Competing interests

The authors declare that they have no competing interests.

## Authors' contributions

CS contributed to the design and implementation of the project, performance evaluation, and analysis, as well as to the preparation of the manuscript. SB contributed to the conception and design of the project and evaluation methods, acquisition of data, interpretation of results, as well as manuscript content. ZT implemented a peak detection system, applied it to the ChIP-seq data set, and interpreted the results. PS designed and implemented a system for visualizing ChIP-seq tags and TSS on the genome. DFH contributed to the conception and overall design of the project, combinational fusion methods, evaluation and interpretation of results, and manuscript content.

## Author information

CS is currently in the Division of Computer Science, Mathematics and Science at St. John's University.

## References

[B1] Sanger F Nicklen S Coulson AR DNA sequencing with chain-terminating inhibitors Proc Natl Acad Sci USA 1977 74 5463 5467 10.1073/pnas.74.12.5463 271968 PMC431765

[B2] Mardis ER The impact of next-generation sequencing technology on genetics Trends Genet 2008 24 3 133 41 10.1016/j.tig.2007.12.007 18262675 PMC2680276

[B3] Shendure J Ji H Next-generation DNA sequencing Nat Biotechnol 2008 26 1135 1145 10.1038/nbt1486 18846087

[B4] Solomon MJ Larsen PL Varshavsky A Mapping protein-DNA interactions in vivo with formaldehyde: evidence that histone H4 is retained on a highly transcribed gene Cell 1988 53 6 937 47 10.1016/S0092-8674(88)90469-2 2454748

[B5] Huebert DJ Kamal M O'Donovan A Bernstein BE Genome-wide analysis of histone modifications by ChiP-on-chip Methods 2006 40 4 365 9 10.1016/j.ymeth.2006.07.032 17101450

[B6] Kim TH Barrera LO Ren B ChIP-chip for genome-wide analysis of protein binding in mammalian cells Curr Protoc Mol Biol 2007 Chapter 21 Unit 21.13 18265397 10.1002/0471142727.mb2113s79

[B7] Johnson DS Mortazavi A Myers RM Wold B Genome-wide mapping of in vivo protein-DNA interactions Science 2007 316 5830 1497 502 10.1126/science.1141319 17540862

[B8] Li H Ruan J Durbin R Mapping short DNA sequencing reads and calling variants using mapping quality scores Genome Res 2008 18 11 1851 1858 10.1101/gr.078212.108 18714091 PMC2577856

[B9] Langmead B Trapnell C Pop M Salzberg SL Ultrafast and memory-efficient alignment of short DNA sequences to the human genome Genome Biol 2009 10 3 R25 10.1186/gb-2009-10-3-r25 19261174 PMC2690996

[B10] Brown S Hsu DF Schweikert C Tang Z Juan HF, Huang HC ChIP-Seq Analytics: Methods and Systems to Improve ChIP-Seq Peak Identification Systems Biology: Applications in Cancer-Related Research 2012 World Scientific Publishing

[B11] Wilbanks EG Facciotti MT Evaluation of algorithm performance in ChIP-seq peak detection PLoS ONE 2010 5 7 e11471 10.1371/journal.pone.0011471 20628599 PMC2900203

[B12] Blahnik KR Dou L O'Geen H McPhillips T Xu X Sole-Search: an integrated analysis program for peak detection and functional annotation using ChIP-seq data Nucleic Acids Res 2009 38 e13 19906703 10.1093/nar/gkp1012 PMC2817454

[B13] Boyle AP Guinney J Crawford GE Furey TS F-Seq: A Feature Density Estimator for High-Throughput Sequence Tags Bioinformatics 2008 24 2537 2538 10.1093/bioinformatics/btn480 18784119 PMC2732284

[B14] Chen X Xu H Yuan P Fang F Huss M Integration of external signaling pathways with the core transcriptional network in embryonic stem cells Cell 2008 133 1106 1117 10.1016/j.cell.2008.04.043 18555785

[B15] Fejes AP Robertson G Bilenky M Varhol R Bainbridge M Jones SJ FindPeaks 3.1: a tool for identifying areas of enrichment from massively parallel short-read sequencing technology Bioinformatics 2008 24 15 1729 30 10.1093/bioinformatics/btn305 18599518 PMC2638869

[B16] Ji H Jiang H Ma W Johnson D Myers R An integrated software system for analyzing ChIP-chip and ChIP-seq data Nat Biotechnol 2008 26 1293 1300 10.1038/nbt.1505 18978777 PMC2596672

[B17] Jothi R Cuddapah S Barski A Cui K Zhao K Genome-wide identification of in vivo protein-DNA binding sites from ChIP-Seq data Nucleic Acids Res 2008 36 5221 5231 10.1093/nar/gkn488 18684996 PMC2532738

[B18] Kharchenko PV Tolstorukov MY Park PJ Design and analysis of ChIP-seq experiments for DNA-binding proteins Nat Biotechnol 2008 26 12 1351 9 10.1038/nbt.1508 19029915 PMC2597701

[B19] Lun DS Sherrid A Weiner B Sherman DR Galagan JE A blind deconvolution approach to high-resolution mapping of transcription factor binding sites from ChIP-seq data Genome Biol 2009 10 R142 10.1186/gb-2009-10-12-r142 20028542 PMC2812949

[B20] Nix DA Courdy SJ Boucher KM Empirical methods for controlling false positives and estimating confidence in ChIP-Seq peaks BMC Bioinformatics 2008 9 523 10.1186/1471-2105-9-523 19061503 PMC2628906

[B21] Qin S Shen J HPeak: A HMM-based algorithm for defining read-enriched regions from massive parallel sequencing data 2009 http://www.sph.umich.edu/csg/qin/HPeak 10.1186/1471-2105-11-369 PMC2912305 20598134

[B22] Spyrou C Stark R Lynch AG Tavare S BayesPeak: Bayesian analysis of ChIP-seq data BMC Bioinformatics 2009 10 299 10.1186/1471-2105-10-299 19772557 PMC2760534

[B23] Valouev A Johnson DS Sundquist A Medina C Anton E Genome-wide analysis of transcription factor binding sites based on ChIP-Seq data Nat Methods 2008 5 829 834 10.1038/nmeth.1246 19160518 PMC2917543

[B24] Zhang Y Liu T Meyer CA Eeckhoute J Johnson DS Bernstein BE Nussbaum C Myers RM Brown M Li W Liu XS Model-based Analysis of ChIP-Seq (MACS) Genome Biology 2008 9 R137 10.1186/gb-2008-9-9-r137 18798982 PMC2592715

[B25] Mortazavi A Williams BA McCue K Schaeffer L Wold B Mapping and quantifying mammalian transcriptomes by RNA-Seq Nat Methods 2008 5 621 628 10.1038/nmeth.1226 18516045 PMC13303166

[B26] Rozowsky J Euskirchen G Auerbach RK Zhang ZD Gibson T PeakSeq enables systematic scoring of ChIP-seq experiments relative to controls Nat Biotechnol 2009 27 66 75 10.1038/nbt.1518 19122651 PMC2924752

[B27] Pepke S Wold B Mortazavi A Computation for ChIP-seq and RNA-seq studies Nat Methods 2009 6 S22 32 10.1038/nmeth.1371 19844228 PMC4121056

[B28] Laajala TD Raghav S Tuomela S Lahesmaa R Aittokallio T Elo LL A practical comparison of methods for detecting transcription factor binding sites in ChIP-seq experiments BMC Genomics 2009 10 618 10.1186/1471-2164-10-618 20017957 PMC2804666

[B29] NYU Center for Health Informatics and Bioinformatics (CHIBI), 2012 http://www.nyuinformatics.org/research/labs/seqinfo

[B30] Hsu DF Chung YS Kristal BS Hsu HH Combinatorial fusion analysis: methods and practice of combining multiple scoring systems Advanced Data Mining Technologies in Bioinformatics 2006 Idea Group Inc 32 36

[B31] Tchou-Wong KM Kiok K Tang Z Kluz T Arita A Effects of Nickel Treatment on H3K4 Trimethylation and Gene Expression PLoS ONE 2011 6 3 e17728 10.1371/journal.pone.0017728 21455298 PMC3063782

[B32] Ho TK Hull JJ Srihari SN Decision combination in multiple classifier system IEEE Trans on Pattern Analysis and Machine Intelligence 1994 16 1 66 75 10.1109/34.273716

[B33] Hsu DF Taksa I Comparing rank and score combination methods for data fusion in information retrieval Information Retrieval 2005 8 3 449 480 10.1007/s10791-005-6994-4

[B34] Hsu DF Kristal BS Schweikert C Rank-Score Characteristics (RSC) Function and Cognitive Diversity Brain Informatics 2010 42 54

[B35] Chuang HY Liu HF Brown S McMunn-Coffran C Kao CY Hsu DF Identifying significant genes from microarray data Proceedings of IEEE Bioinformatics and Bioengineering 2004 2004 IEEE Computer Society 358 365

[B36] Peng CH Hsu JT Chung YS Lin YJ Chow WY Hsu DF Tang CY Identification of degenerate motifs using position restricted selection and hybrid ranking combination Nucleic Acids Research 2006 34 22 6379 6391 10.1093/nar/gkl658 17130169 PMC1702486

[B37] Lin KL Lin CY Huang CD Chang HM Yang CY Lin CT Tang CY Hsu DF Feature combination criteria for improving Accuracy in protein structure prediction IEEE Transactions on NanoBioscience 2007 6 2 186 196 17695755 10.1109/tnb.2007.897482

[B38] Yang JM Chen YF Shen TW Kristal BS Hsu DF Consensus scoring for improving enrichment in virtual screening J Chem Inf Model 2005 45 1134 1146 10.1021/ci050034w 16045308

[B39] Ng KB Kantor PB Predicting the effectiveness of naive data fusion on the basis of system characteristics J Am Soc Inform Sci 2000 51 12 1177 1189

[B40] Lyons DM Hsu DF Combining multiple scoring systems for target tracking using rank-score characteristics Information Fusion 2009 10 2 124 136 10.1016/j.inffus.2008.08.009

[B41] Schweikert C Liu J An W Brown S Smith PR Combining Multiple Detection Systems for Improving ChIP-seq Peak Identification of Protein Binding Sites Department of Computer and Information Science Technical Report 11-2010 2010 Fordham University, New York, NY

